# The use of fluorescence *in situ* hybridization techniques in the detection of microdeletion syndromes

**DOI:** 10.1186/1471-2164-15-S2-P60

**Published:** 2014-04-02

**Authors:** Muna M ALmughamsi, Taha A Kumosani, Emad A ALhamzi, Mohammed Al-Qahtani

**Affiliations:** 1 Center of Excellence in Genomic Medicine Unit, King Abdulaziz University, Jeddah, Saudi Arabia; 2 Faculty of Science, Biochemistry Department, King Abdulaziz University, Jeddah, Saudi Arabia

## Background

Microdeletion syndromes are a heterogenous group of disorder caused by the deletion of specific regions of chromosomal DNA causing haplo insufficiencies for important genes [1]. These deletions are difficult to visualize using standard cytogenetic techniques. Fluorescence in situ hybridization (FISH) can resolve these submicroscopic deletions to a lower limit of approximately 3MB and has therefore become the method of choice for the diagnosis of these disorders. Deoxyribonucleic acid (DNA) FISH probes can be used in metaphase and interphase cells to detect these specific regions of deletion probes [2]. The region deleted is known as typically deleted region (TDR) or critical region. There are different microdeletion syndromes such as Prader-Willi/Angelman syndrome, William’s, DiGeorge, Smith- Magenis and Miller-Dieker syndromes. Of these, FISH probes for Prader-Willi /Angelman, William’s and DiGeorge syndromes are currently available in our laboratory. This study aimed to compare between cultured and uncultured peripheral blood using Fluorescence in situ hybridization technique for the detection of microdeletion syndromes and to study the concordance rate between the two methods.

## Materials and methods

In the current study, 50 subjects clinically diagnosed as patients of microdeletion syndromes were collected randomly from different ages. Peripheral blood containing lymphocytic cells was cultured for 72 hours using culture media in the presence of phytohemagglutinin (PHA) to provide sufficient metaphase nuclei for analysis. After culturing and harvesting the metaphases, slide preparation and FISH techniques assay were performed.

## Results

The result of both cultured and uncultured FISH showed that out of 50 patients, 6 were positive and 44 were negative for microdeletion syndrome. In the case of Praderwilli/Angelman syndrome out of 21 patients, all (100%) gave negative result. In the case of DiGeorge syndrome out of 19 cases, 2(10%) gave positive result and the rest 17(90%) gave negative result. For William’s syndrome out of total 10 cases, 4(40%) gave positive result and 6 (60%) gave negative result.

## Conclusions

In conclusion, interphase from uncultured FISH is rapid, reliable, cost effective and shows same result as metaphase from culture FISH. The interphase FISH is especially suitable for medical urgent cases.
